# Understanding the role of potential pathways and its components including hypoxia and immune system in case of oral cancer

**DOI:** 10.1038/s41598-021-98031-7

**Published:** 2021-10-01

**Authors:** Leena Hussein Bajrai, Sayed Sartaj Sohrab, Mohammad Mobashir, Mohammad Amjad Kamal, Moshahid Alam Rizvi, Esam Ibraheem Azhar

**Affiliations:** 1 Special Infectious Agents Unit, King Fahd Medical Research Centre, King Abdulaziz University, Jeddah, Saudi Arabia; 2 Biochemistry Department, Faculty of Sciences, King Abdulaziz University, Jeddah, Saudi Arabia; 3 Medical Laboratory Sciences Department, Faculty of Applied Medical Sciences, King Abdulaziz University, Jeddah, Saudi Arabia; 4 Department of Microbiology, Tumor and Cell Biology (MTC) Karolinska Institute, Novels väg 16, Solna, 17165 Stockholm, Sweden; 5 West China School of Nursing / Institutes for Systems Genetics, Frontiers Science Center for Disease-Related Molecular Network, West China Hospital, Sichuan University, Chengdu, 610041 Sichuan China; 6 King Fahd Medical Research Center, King Abdulaziz University, P. O. Box 80216, Jeddah, 21589 Saudi Arabia; 7 Enzymoics, Novel Global Community Educational Foundation, 7 Peterlee Place, Hebersham, NSW 2770 Australia; 8 The Genome Biology Lab, Department of Biosciences, Jamia Millia Islamia, New Delhi, 110025 India; 9 SciLifeLab, Department of Oncology and Pathology, Karolinska Institutet, P. O. Box 1031, 17121 Stockholm, Sweden

**Keywords:** Cancer genomics, Oral cancer, Computational biology and bioinformatics, Immunology

## Abstract

There are a few biological functions or phenomenon which are universally associated with majority of the cancers and hypoxia and immune systems are among them. Hypoxia often occurs in most of the cancers which helps the cells in adapting different responses with respect to the normal cells which may be the activation of signaling pathways which regulate proliferation, angiogenesis, and cell death. Similar to it, immune signaling pathways are known to play critical roles in cancers. Moreover, there are a number of genes which are known to be associated with these hypoxia and immune system and appear to direct affect the tumor growth and propagations. Cancer is among the leading cause of death and oral cancer is the tenth-leading cause due to cancer death. In this study, we were mainly interested to understand the impact of alteration in the expression of hypoxia and immune system-related genes and their contribution to head and neck squamous cell carcinoma. Moreover, we have collected the genes associated with hypoxia and immune system from the literatures. In this work, we have performed meta-analysis of the gene and microRNA expression and mutational datasets obtained from public database for different grades of tumor in case of oral cancer. Based on our results, we conclude that the critical pathways which dominantly enriched are associated with metabolism, cell cycle, immune system and based on the survival analysis of the hypoxic genes, we observe that the potential genes associated with head and neck squamous cell carcinoma and its progression are STC2, PGK1, P4HA1, HK1, SPIB, ANXA5, SERPINE1, HGF, PFKM, TGFB1, L1CAM, ELK4, EHF, and CDK2.

## Introduction

Hypoxia often occurs in cancer and helps the cells in adapting different responses than the normal cells such as the triggering of signaling pathways regulating critical biological processes (proliferation, angiogenesis, and cell death or apoptosis)^1^. So far from the previous work, a number of genes associated with these processes and functions have been explored and investigated. Similarly, different forms of cancer require different immune systems and associated signalling pathways, and the immune signalling network (ISN) may be a major component in cancer genesis and progression. Although it has been established that cancers, including head and neck cancer, are immunogenic tumours for which immunotherapy is aggressively sought by targeting immunological checkpoints, an immune-based prognostic signature remains a viable option^2–4^. Several prior works^2–4^ propose pathway-level knowledge and analytic methodologies, as well as Hansen and Iyengar’s^4^ computational strategy to bridge the gap between precision medicine and systems treatments. Comprehension and unravelling comprehensive and minute understanding of cell phenotypes and disease pathophysiology remains a basic problem, as do the molecular mechanisms that lead to disease initiation and oral cancer progression.

Oral cancer is the 10th most prevalence cancer globally and in general classified as head and neck squamous cell carcinoma (HNSCC). It is a malignant neoplasia which arises in oral cavity of lip, tongue, gingiva, mouth floor and glands^5–7^ which may originate by a number of factors such as genetic alterations, gene expression alterations, and mutations^5,8–13^. Furthermore, more factors may act as the potential cause of such cancer and one of the cause is HPV (Human Pappiloma Virus) infection and this virus is non-enveloped icosahedral capsid with circular double standard DNA which majorly cause cervical cancer in human^12,14,15^. In addition, there exit potential difference between HPV-induced oral cancers than that of HPV-negative (oral) tumors in terms of the clinical response and finally the overall survival rates^16,17^. In the previous study, there are lots of work which have been performed for the study of oral cancer such as mutational, gene, and miRNA expression profiling, epigenetic changes, and proteomics for HNSCC^5,12,18,19^.

When we’re looking for a profound understanding of something, from a computational study to a therapeutic method^20–22^. It provides a ray of hope for a revolutionary diagnostic technique. Thus, in HNSCC, the hypoxia and immune-based prognostic signatures maintain a diagnostic potential that can be further explored and examined. We chose a publically available gene expression dataset for this purpose and evaluated the data with the goal of understanding how signaling networks and their components are relevant to the immune system. In this study, our goal was to understand the impact of alteration in the expression of hypoxia and immune system-related genes and their contribution to head and neck cancer^23,24^. For this purpose, we have collected the hypoxia-associated genes based on the literature related to diverse biological processes and functions and have also collected the mutational and expression (both microRNA and gene) datasets from Gene Expression Omnibus (GEO) freely accessible public database (http://www.ncbi.nlm.nih.gov/geo/) for which we have performed comparative analysis and the clinical relevance i.e., survival analysis^25–27^.

Based on our work, we observe that there are certain sets of genes which are always differentially expressed irrespective of the stages and similar to it there are a number of pathways which are potentially altered in result to the differential gene expression patterns. Based on our results, we conclude that the critical pathways which are dominantly enriched are associated with metabolism, cell cycle, immune system.

## Materials and methods

In the first step, we have selected the data of our interest for mutation, gene, and miRNA expression analysis. For the gene expression and miRNA expression datasets, the samples have been analyzed by using the inbuilt tool GEO2R^28,29^ and the mutational dataset which have been obtained from TCGA database have been analyzed from in-house MATLAB code. For pathway enrichment analysis, the similar protocols have been followed as per DAVID and panther databases^30–33^. For differential gene expression analysis, we have compared the tumor samples with normal samples to generate differentially expressed genes and miRNAs lists and to generate the list of mutated genes the threshold has been set i.e., 5% of the samples showing the mutation for specific genes. After preparing the lists (overexpressed genes and microRNAs) and the mutated genes, we proceed for our goal which is to understand the expression and mutational patterns^10,34^ and its inferred functions^33,34^. All the pathways have p-values less than 0.05, blue color means highest p-values and the yellow color for lowest p-value. For generating DEGs network, FunCoup2.0^35^ has been used for all the networks throughout the work and cytoscape^36^ has been used for network visualization. For most of our coding and calculations MATLAB has been used. FunCoup predicts four different classes of functional coupling or associations such as protein complexes, protein–protein physical interactions, metabolic, and signaling pathways^35^.

## Results

### Gene expression profiling reveals that there are selected sets of pathways are mostly affected as a result of alteration in gene expression

**Figure 1:**
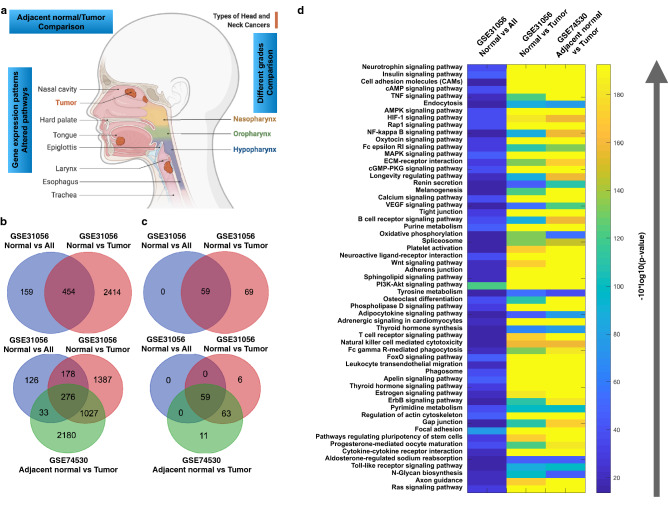
Differential gene expression profiling of different grades of oral cancer. (**a**) Venn diagram for differentially expressed genes, inferred, and enriched pathways; (**b**) Plot to show the overall number of DEGs, inferred, and enriched pathways.

In the first step, the major goal was to understand the gene expression patterns and profiles between different tumor stages for which we have used the oral cancer expression dataset from GEO GSE84846^7^. The dataset contains the samples which have tongue squamous cells carcinoma cells of both male and female of different ages and different stages from stage I to IV. We have performed comparative analysis of grade I with grade II, III, and IV to investigate the evolved DEGs and altered functions from grade I to IV and the total number of samples were 99. Here, we observe that the number of DEGs is comparatively low for grades I and II i.e., 24, for grades I and III is 40 and for grades I and IV is 175 and number of shared genes are quite low (Fig. 1a) and alterations in gene expression pattern increases exponentially from grade I to IV. Irrespective of the tumor location and the number of DEGs there are 59 pathways which are commonly enriched even in combination with the dataset GSE31056 (Fig. 1b,c). All these 59 enriched pathways have been displayed with their respective p-values under different conditions (Fig. 1d) and more details have been presented as Supplementary Data 1. Further, we have also compared this data with another dataset (GSE37991)^37–41^ in Supplementary Data 2. Here, we also observe that both the datasets share a large number of vital DEGs and the pathways.

**Figure 2:**
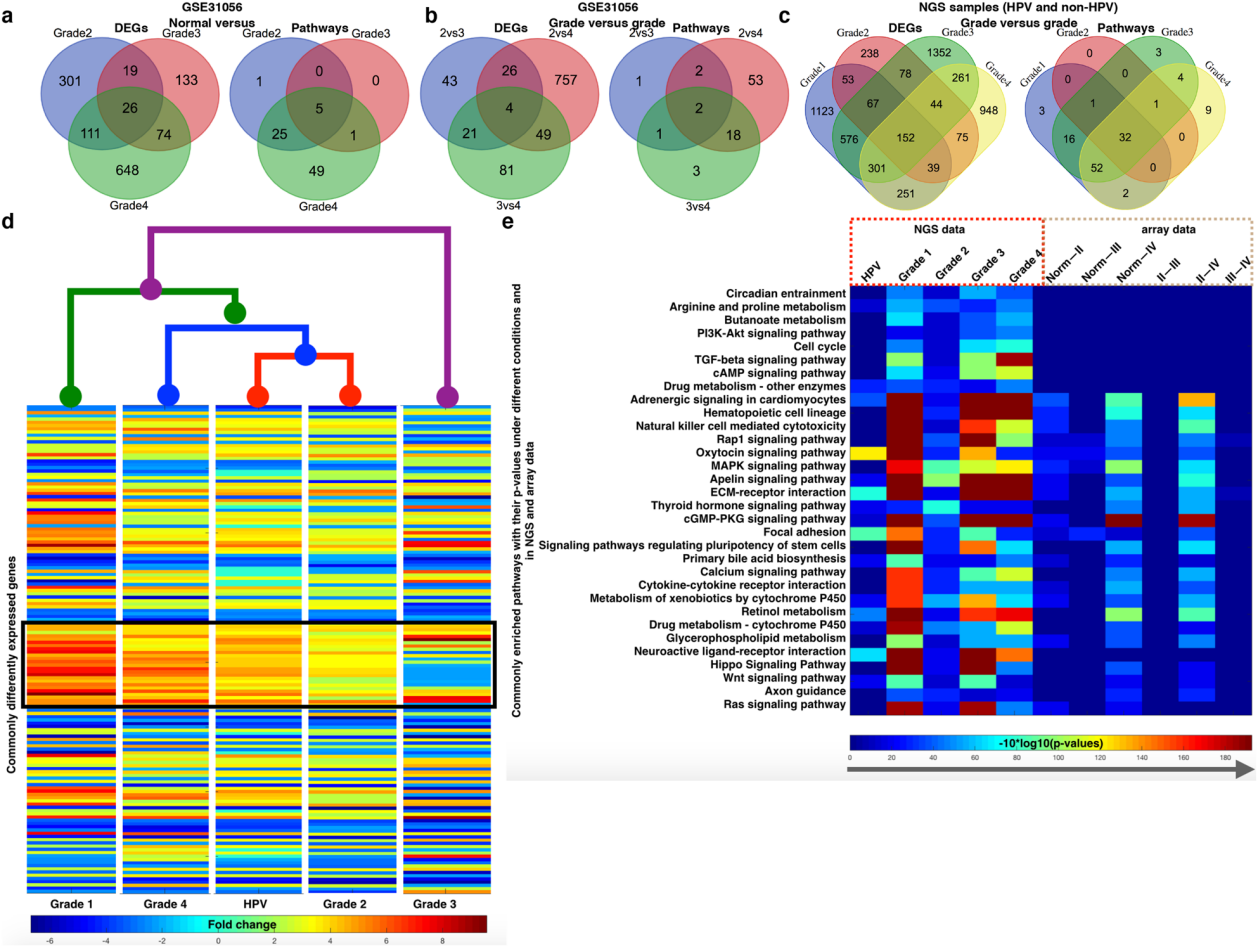
Differentially expressed genes and the enriched pathways for HPV infected oral cancer. Venn diagram for the different combinations of DEGs and the enriched pathways for (**a,b**) GSE31056 and (**c**) NGS dataset. (**d**) Heatmap and cluster for the 152 commonly DEGs in case of NGS data. (**e**) Commonly enriched 32 pathways for NGS dataset and the p-values for these pathways including the array dataset.

We have compared gene expression pattern of overlapped genes in HPV infected samples from grade I–IV (Fig. 2a–c). Here, we tried to present a list of genes (table/supplementary) irrespective of tumor grades, at the same time it also shows dissimilarity in level of gene expression and functional impact. Figure 2d states that Grade I and IV are having similar pattern of the expression, grade II shares their pattern with all four grades of HPV infected tumor whereas grade III showed distinct from all other grades (Fig. 2a–c). It claims that grade II is important stage where all HPV mediated oncogenic components are expressed. In Fig. 2d, we have inserted a box with black line which is just to show the reverse behavior in terms of expression for the selected genes grade 3 versus all other conditions. Overlapped, DEGs of oral tumor also showed difference in functional effect. Figure 2e shows that there are 32 pathways which are mainly altered in HPV-infected oral cancer and those *p*-value were compared with altered pathways of non-infected oral cancer (GEO datasets) pathways, it revealed that few of the pathways such as circadian entrainment, arginine and proline metabolism, butanoate metabolism, PI3K-AKT, cell cycle, TGF-beta, cAMP, neuro active ligand receptor interaction are highly altered in HPV-infected tumor than non-infected oral cancer which implies enhanced vulnerability of HPV infection in oral cancer. Here, we also observe that functional effect and gene expression followed the same pattern (Fig. 2d,e). Thus, we conclude that different grades of oral tumor lead to diverse impact on gene expression pattern and their functions.

### Higher mutations leads to the potential change in critical biological functions associated with oral cancer

**Figure 3:**
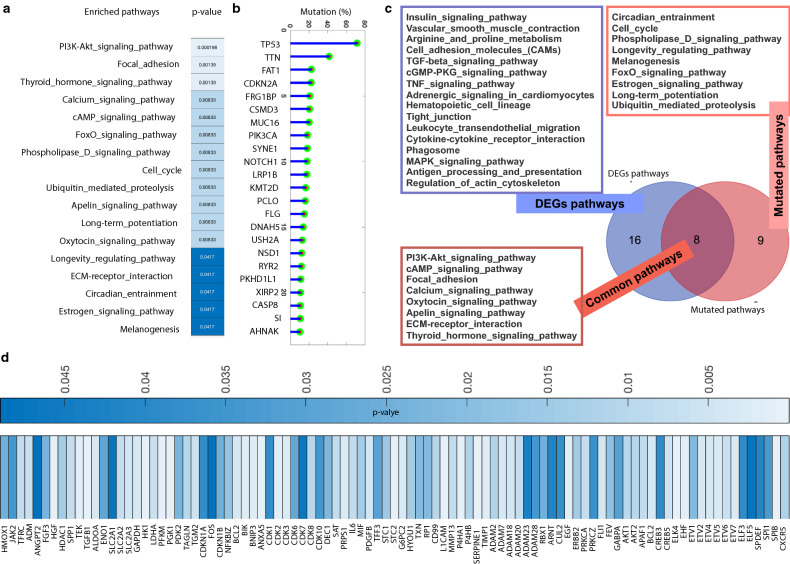
Mutational profiling and functional impact in oral cancer. (**a**) We have performed pathway enrichment analysis for those genes which appear to have more than 5% mutation for the selected dataset from TCGA database; (**b**) Genes with mutations ≥ 10%; (**c**) Comparison of the altered functions with respect to mutations and differential expression; (**d**) p-value for Kaplan-Meyer plots after survival analysis of the hypoxic genes in case of head and neck cancer.

For mutational profiling, we have used the datasets from TCGA database which contains 530 samples (Head and Neck Squamous Cell Carcinoma, Firehose Legacy) and from here, we have selected the mutated genes which appear minimum in 5% of the samples and performed the pathway enrichment analysis where PI3K-Akt signaling, focal adhesion, thyroid hormone signaling, calcium signaling, cAMP signaling, FoxO signaling, phospholipase-d signaling, cell cycle, ubiquitin mediated proteolysis, apelin signaling, long-term potentiation, oxytocin signaling, Longevity regulating, ECM-receptor interaction, circadian entrainment, estrogen signaling, and melanogenesis (Fig. 3a) are among the enriched pathways for the selected genes which were mutated in minimum of the 5% of the samples. Furthermore, the top mutated (≥ 10%) genes and observe that TP53, TTN, FAT1, CDKN2A, FRG1BP, CSMD3, MUC16, PIK3CA, SYNE1, NOTCH1, LRP1B, KMT2D, PCLO, FLG, DNAH5, USH2A, NSD1, RYR2, PKHD1L1, XIRP2, CASP8, SI, and AHNAK (Fig. 3b) are among the highly mutated genes. Majority of these genes are well known to be associated with a number of cancers including the head and neck cancer and the similar case is with the enriched pathways. Furthermore, we have drawn a venn diagram to look over the commonly and specific altered pathways both because of altered expression or mutations (Fig. 3c). PI3K-Akt signaling, cAMP signaling, Focal adhesion, Calcium signaling, Oxytocin signaling, Apelin signaling, ECM-receptor interaction, and thyroid hormone signaling are those pathways which are commonly altered in terms of gene over expression and mutations which gives more significance to these pathways while circadian entrainment, cell cycle, phospholipase-d signaling, longevity regulating pathway, melanogenesis, FoxO signaling, estrogen signaling, long-term potentiation, and ubiquitin mediated proteolysis signaling pathways are specifically altered due to mutation. There are 16 signaling pathways which are exclusively altered due to over expression and some of them are insulin signaling, CAMs, TGF, TNF, tight junction, cGMP-PKG, phagosome signaling pathways and more.

**Figure 4:**
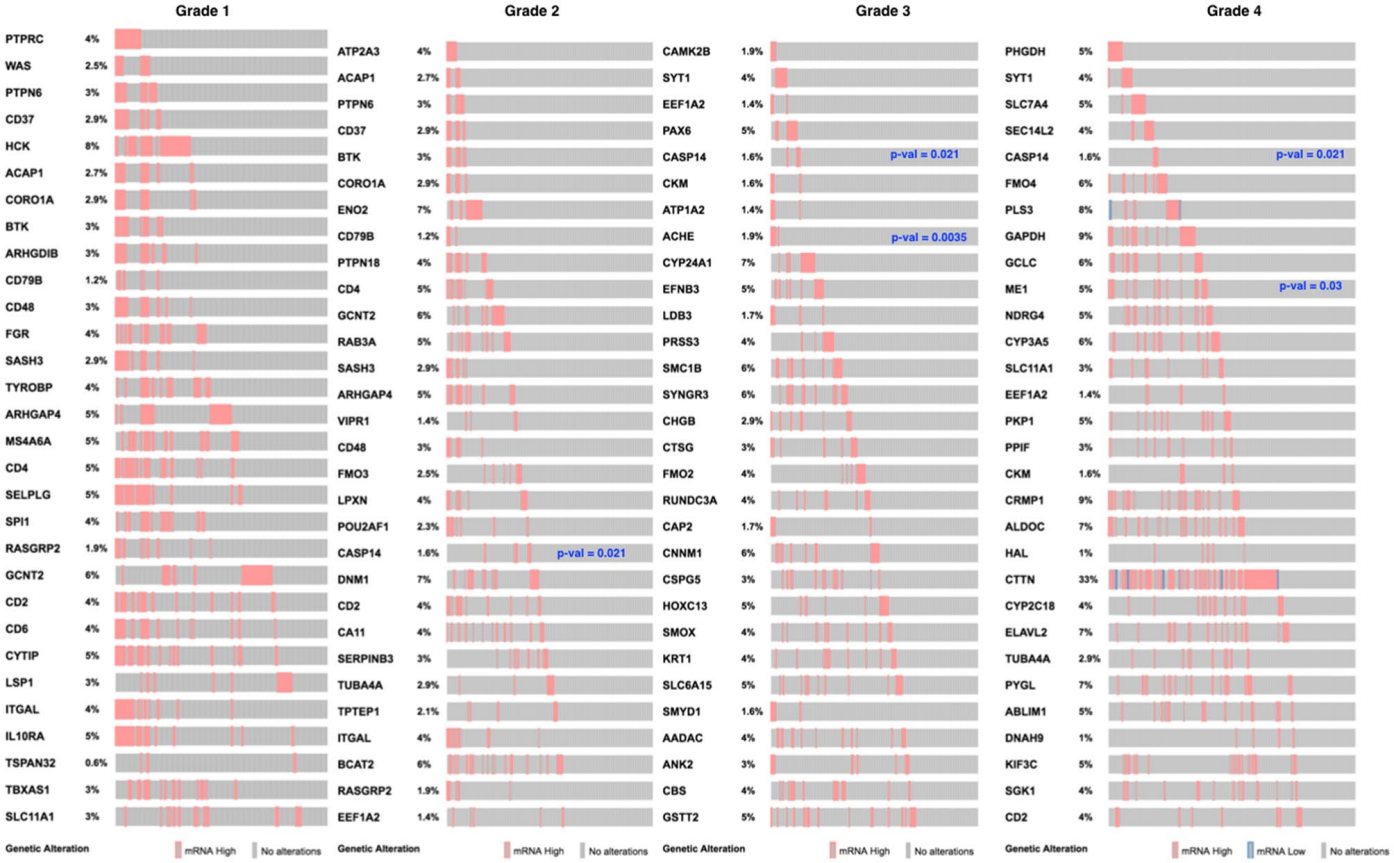
Clinical relevance. Clinical Relevance for the top-ranked genes (based on connectivity of the genes within the network generated through network database) and respective inferred pathways. p-value represents the clinical significance in terms of survival analysis and the TCGA database and cBioPortal have been used.

Furthermore, we have also investigated the clinical relevance of the hypoxic genes by performing survival analysis (Kaplan-Meyer plot) and plot the heatmap of the p-values of all those genes (Fig. 3d) which appear significant (p-value < 0.05). Overall, 95 genes appear significant in case of head and neck cancer and STC2, PGK1, P4HA1, HK1, SPIB, ANXA5, SERPINE1, HGF, PFKM, TGFB1, L1CAM, ELK4, EHF, and CDK2 appear to be highly significant which have p-values even lower than or equal to 0.0013. After analyzing the clinical relevance, we have fetched the inferred pathways for these top-ranked genes and we observe that these genes not only relate with the hypoxic condition but also a number of fully those pathways which directly affect the tumor initiation, propagation, and growth as shown in Table 1. Moreover, these top-ranked genes have been processed for detailed clinical relevance for which their overexpression have been checked in patients samples in TCGA database (Fig. 4) for all the four grades and the percentage of the patients with overexpressed genes have been shown.

**Table 1:** Top hypoxic genes with p-values ≤ 0.0013 and the associated pathways.

Genes	Pathways
HGF	Cytokine-cytokine_receptor_interaction
HGF	Focal_adhesion
HGF	Pathways_in_cancer
HGF	Renal_cell_carcinoma
HGF	Melanoma
PGK1	Glycolysis_/_gluconeogenesis
PGK1	Carbon_fixation_in_photosynthetic_organisms
TGFB1	MAPK_signaling_pathway
TGFB1	Cytokine-cytokine_receptor_interaction
TGFB1	Cell_cycle
TGFB1	TGF-beta_signaling_pathway
TGFB1	Leishmaniasis
TGFB1	Chagas_disease
TGFB1	Pathways_in_cancer
TGFB1	Colorectal_cancer
TGFB1	Renal_cell_carcinoma
TGFB1	Pancreatic_cancer
TGFB1	Chronic_myeloid_leukemia
SERPINE1	p53_signaling_pathway
SERPINE1	Complement_and_coagulation_cascades
P4HA1	Arginine_and_proline_metabolism
CDK2	Cell_cycle
CDK2	Oocyte_meiosis
CDK2	p53_signaling_pathway
CDK2	Progesterone-mediated_oocyte_maturation
CDK2	Prostate_cancer
CDK2	Small_cell_lung_cancer
PFKM	Glycolysis_/_gluconeogenesis
PFKM	Pentose_phosphate_pathway
PFKM	Fructose_and_mannose_metabolism
PFKM	Galactose_metabolism
PFKM	Insulin_signaling_pathway
HK1	Glycolysis_/_gluconeogenesis
HK1	Fructose_and_mannose_metabolism
HK1	Galactose_metabolism
HK1	Starch_and_sucrose_metabolism
HK1	Amino_sugar_and_nucleotide_sugar_metabolism
HK1	Streptomycin_biosynthesis
HK1	Insulin_signaling_pathway
HK1	Type_II_diabetes_mellitus
ELK4	MAPK_signaling_pathway
L1CAM	Axon_guidance
L1CAM	Cell_adhesion_molecules_(CAMs)
TGFB1	Hippo_Signaling_Pathway
SERPINE1	Hippo_Signaling_Pathway
HGF	Ras_signaling_pathway
HGF	Rap1_signaling_pathway
SERPINE1	Apelin_signaling_pathway
HK1	HIF-1_signaling_pathway
SERPINE1	HIF-1_signaling_pathway
PGK1	HIF-1_signaling_pathway
CDK2	FoxO_signaling_pathway
TGFB1	FoxO_signaling_pathway
CDK2	PI3K-Akt_signaling_pathway
HGF	PI3K-Akt_signaling_pathway
PFKM	AMPK_signaling_pathway
TGFB1	Osteoclast_differentiation

### Differential microRNAs expression also potentially impact the cancer associated functions

**Figure 5:**
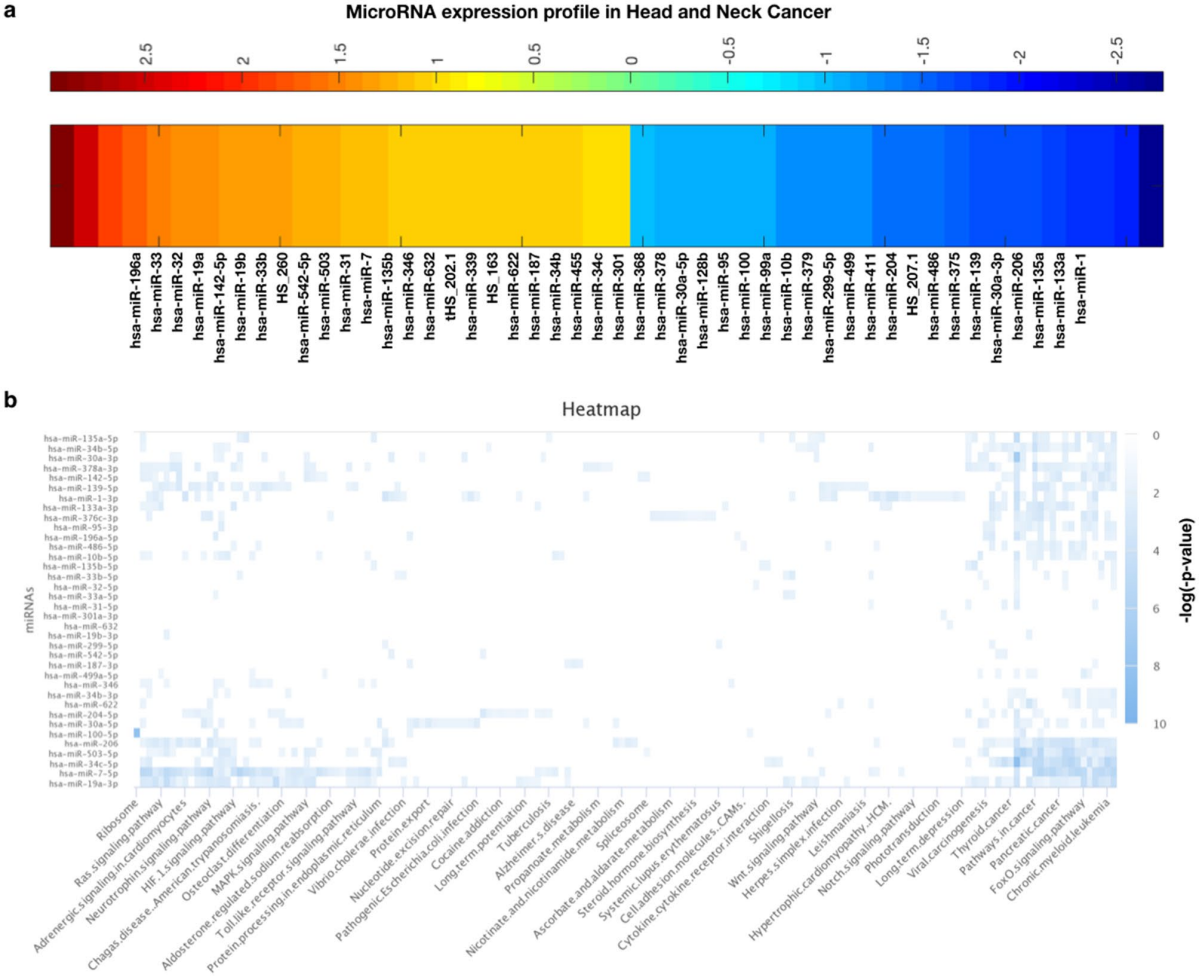
miRNA expression profiling and their functional impact in head and neck cancer. (**a**) Differentially expressed miRNAs and (**b**) the enriched pathways with the respective miRNAs.

After analyzing the gene expression and mutational profiling, we have performed miRNA expression profiling and for this purpose, the dataset was collected from GEO database i.e., GSE31227 and the platform was GPL9770^19^. This dataset contains 15 patient surgical margin as controls and 15 patient Oral Squamous Cell Carcinoma (OSCC) and we found that there are 46 miRNAs out of 739 miRNAs which are overexpressed in case of head and neck cancer (Fig. 5a) and in terms of functions the most affected biological pathways are thyroid cancer, pathways in cancer, pancreatic cancer, Foxo signaling, chronic myeloid leukemia, HIF1 signaling and more and most of these pathways appears to strongly associated with head and neck cancer and oral cancer (Fig. 5b). Here, it can be clearly seen that the pathways enriched in DEGs and the miRNA pathways list have a number of common pathways such as HIF-1 signaling, Ras, MAPK, immune system associated pathways, and directly cancer-associated pathways.

## Discussion

Hypoxia and ISN often help the cells in adapting different responses than the normal cells such as the triggering of signaling pathways regulating critical biological processes (proliferation, angiogenesis, and cell death or apoptosis)^15,20,42–49^. These two processes have been explored in a number of human diseases and different from the previous works, here these two systems have been explored simultaneously in HNSCC and also the role of microRNAs have been analyzed with their effect on biological processes and functions and furthermore the survival analysis^12,50–52^ has been analyzed for the hypoxic genes. The reason to focus on hypoxia and mainly the ISN was that the associated signaling pathways with these two systems are considered as a master regulator for cancer initiation and progression and has also been proven that HNSCC is an immunogenic tumor and immunotherapy is strongly pursued through targeting on the immune checkpoints the immune based prognostic signature remains a potential that can be applied^2–4^. In the previous works^2–4^, pathway-level understanding and analysis approaches has also been presented which present computational approach to bridge between precision medicine and systems therapeutics.

In this study, the main focus of the study was to understand the expression pattern of both the genes and the microRNAs and the mutational profiling followed by the survival analysis for HNSCC for which the datasets have been utilized from the GEO and the TCGA database. After the expression and mutational profiling, we performed comparative analysis for the functions in both the cases. PI3K-Akt signaling, cAMP signaling, focal adhesion, calcium signaling, oxytocin signaling, apelin signaling, ECM-receptor interaction, and thyroid hormone signaling are those pathways which are commonly enriched for both the cases differential expression and mutation in HNSCC which gives higher significance to these pathways for the selected disease while there are specific pathways for DEGs and mutated genes lists which means there are pathways which may be altered only because of overexpression of the genes or higher mutations rate. Mutation-specific altered pathways are circadian entrainment, cell cycle, phospholipase-d signaling, longevity regulating pathway, melanogenesis, FoxO signaling, estrogen signaling, long-term potentiation, and ubiquitin mediated proteolysis signaling pathways while insulin signaling, CAMs, TGF, TNF, tight junction, cGMP-PKG, phagosome signaling pathways are altered gene expression specific. From OSCC microRNAs analysis, 46 miRNAs appear overexpressed and the most affected functions are thyroid cancer, pathways in cancer, pancreatic cancer, Foxo signaling, chronic myeloid leukemia, HIF1 signaling and more and most of these pathways appears to strongly associated with head and neck cancer and oral cancer. Based on the clinical relevance of the hypoxic genes, there are a large number of genes which are highly significant and STC2, PGK1, P4HA1, HK1, SPIB, ANXA5, SERPINE1, HGF, PFKM, TGFB1, L1CAM, ELK4, EHF, and CDK2 are highly significant which have p-values even lower than or equal to 0.0013. Similar to the expression and mutational profiling, the inferred pathways of the top-ranked genes are direct components of those pathways which directly affect the tumor initiation, propagation, and growth. Moreover, the RNA and miRNA expression analysis shows that there are common functions in the RNA and the miRNA pathways list such as HIF-1 signaling, Ras, MAPK, immune system associated pathways, and directly cancer-associated pathways.

## Conclusions

As mentioned that the major goal of this study was to understand the role of expression profiling of genes and the microRNAs and the mutational profiling of the genes and also the clinical relevance in case of HNSCC and based on results and the analysis, it leads to the conclusion that the critical pathways which could be dominantly enriched or altered in case of HNSCC are associated with metabolism, cell cycle, immune system, and hypoxia and the three different datasets of gene expression and microRNA expression, and the mutational data also leads to the conclusion that the pathways and the pathways components potentially associated with HNSCC and its progression.

## Supplementary Information

Supplementary Information 1.

Supplementary Information 2.
