# Supplementary material for: Understanding the role of potential pathways and its components including hypoxia and immune system in case of oral cancer
Source: Sci Rep. 2021 Oct 1;11:19576. doi: 10.1038/s41598-021-98031-7 (PMC8486818; doi:10.1038/s41598-021-98031-7)
Supplement: Supplementary file 2 — Supplementary Information 2. [file 41598_2021_98031_MOESM2_ESM.pdf]

Supplementary data 2

Compared with previous data

DEGs

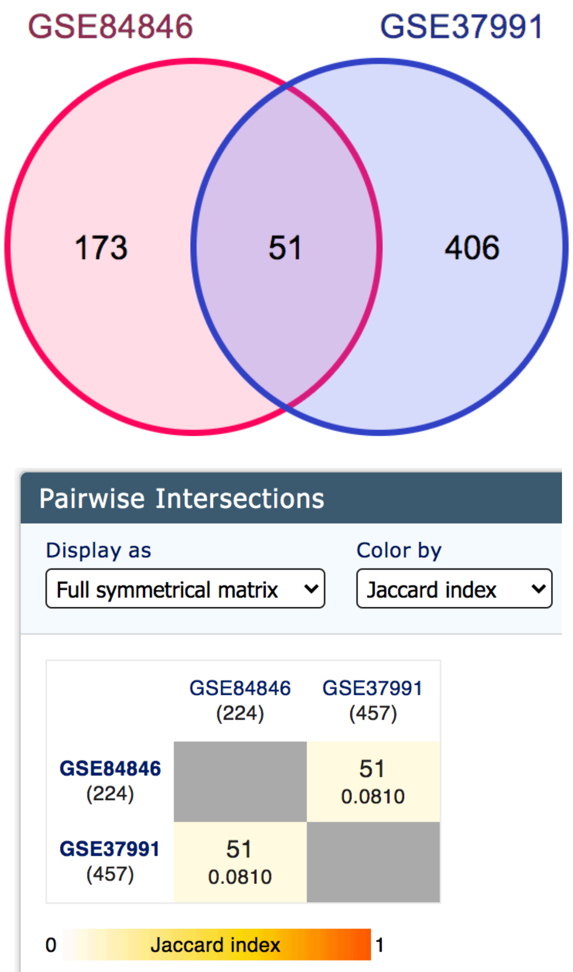

Enriched pathways

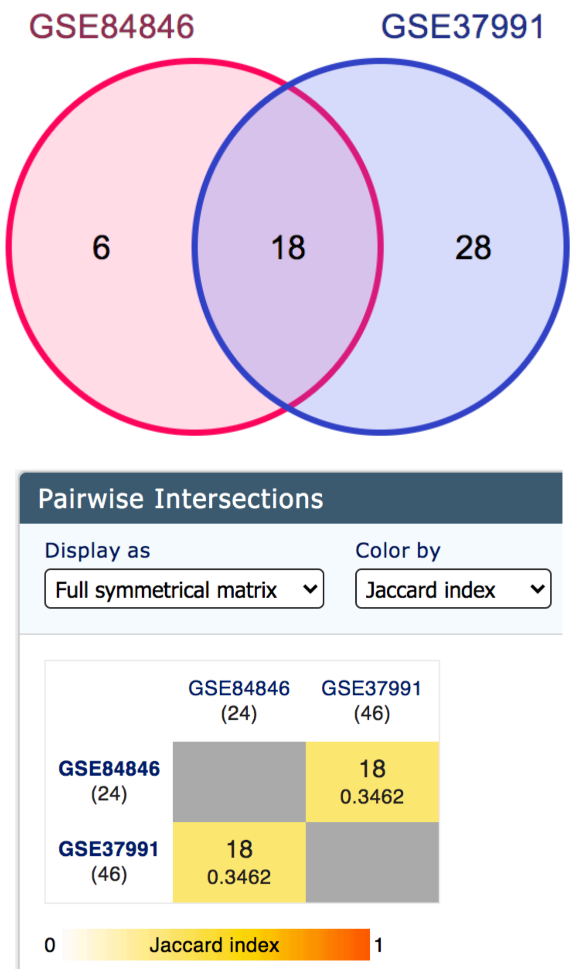

https://www.molbiotools.com/listcompare.php

Supplementary data 2. Cross-verification of the dataset for better clarity. Both the datasets belongs to oral cancer. The differentially expressed genes and the enriched pathways have been compared by using venn diagram. The link for plotting venn diagram is mentioned above.
